# Histone methyltransferase enhancer of zeste 2 polycomb repressive complex 2 subunit exacerbates inflammation in depression rats by modulating microglia polarization

**DOI:** 10.1080/21655979.2022.2036892

**Published:** 2022-02-17

**Authors:** Xuezhu Huang, Qin Yang, Lingling Xie, Sihong Lei

**Affiliations:** a Mental Medicine, College of Wenzhou Medical University, Wenzhou, Zhejiang, China; b Department of Psychosomatic Medicine, Nanchong Central Hospital Affiliated with North Sichuan Medical College, Nanchong, Sichuan, China; c Department of Geriatrics, Kangning Hospital Affiliated with Wenzhou Medical University, Wenzhou, Zhejiang, China

**Keywords:** Depression, microglia, enhancer of zeste homolog 2, microRNA-29b-3p, MMP2, neuroinflammation

## Abstract

Depression is a major cause of emotional agony and degraded living quality. Enhancer of zeste 2 polycomb repressive complex 2 subunit (EZH2) is involved in histone methylation in human diseases. This experiment was designed to investigate the mechanism of EZH2 on depression. Depression rat model was established via the treatment of chronic unpredictable mild stress (CUMS) to identify rat depression-like behaviors. EZH2 expression was determined and then silenced to assess its effect on depression-like behaviors and neuroinflammation. Microglia were isolated, cultured, identified and activated to assess EZH2 expression. Effect of EZH2 on microglia polarization was evaluated. Next, the binding relation between microRNA (miR)-29b-3p and EZH2 or matrix metallopeptidase 2 (MMP2) was analyzed. Levels of miR-29b-3p expression and MMP2 transcription were examined. Additionally, the role of miR-29b-3p in microglia polarization was tested. Depression-like behaviors were exhibited after CUMS induction. EZH2 was overexpressed in CUMS-treated rats and lipopolysaccharide (LPS)-induced microglia. EZH2 silencing reversed depression-like behaviors. EZH2 silencing mitigated inflammation in depression by manipulating microglia M2-type polarization. EZH2 targeted miR-29b-3p expression to promote MMP2 transcription. Inhibition of miR-29b-3p reversed the role of EZH2 silencing **in** microglia M2-type polarization and promoted inflammation. EZH2 inhibited miR-29b-3p expression by combining with miR-29b-3p promoter and trimethylation of histone H3-lysine 27-trimethylated upregulation, and then elevated MMP2 transcription and triggered microglia M1-type polarization, thus exacerbating depression-like behaviors and neuroinflammation of depression.

## GRAPHICAL ABSTRACT

**Figure:**
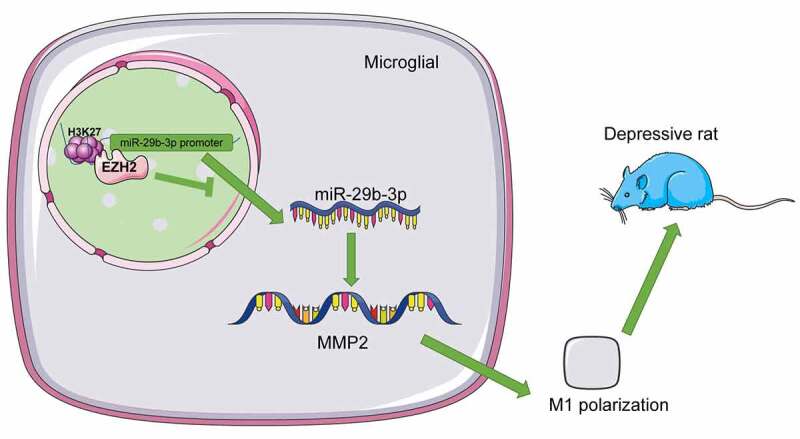

## Introduction

Numerous medical advances have corroborated that depression is, instead of an exclusive mood disorder to female at middle age, a severe global healthy burden in different socioeconomic classes that impairs psychosocial capabilities and living quality [1,2]. The major incentives in depression include stressors, cognition progression, inheritance, behavioral customs and sociodemographic causes, which are all hereditarily or biologically associated with depression [2]. Besides, depression is tightly related to the interaction between inflammatory symptoms and brain neural circuits [3]. As a predominant chronic mental disorder featured by insomnia, melancholy and lack of enthusiasm or energy, depression also destructively impacts mood, thought and even the state of body health [4]. Although psychological interventions, behavioral activation, drug administration, and cognitive behavior therapy could bring some therapeutic benefits to depression patients, they could neither permanently cure depression, nor its complications [5,6]. Against this backdrop, novel therapeutic strategies for depression are urgently required.

Activated microglia in tissues of depression patients elicit neuroinflammation, thereby accelerating neuron cellular loss and disrupting brain functions to encourage depression-like behaviors [7]. Moreover, microglia polarization is a classical mechanism in depression progression as it is divided into M1 (inflammatory effect) and M2 (anti-inflammatory effect) type to impair or protect subjects with depression [8]. Enhancer of zeste homolog 2 (EZH2), a kind of epigenetic participator in histone methylation, retards neuronal activities and induces depression-like behaviors in forebrain of mice [9]. EZH2 catalyzes inflammatory infiltration and microglial activation in depression development [10]. Thence, EZH2 might be involved in depression via modulating microglia polarization.

Notably, inhibition of EZH2 upregulates microRNA (miR)-29b expression by suppressing the protein level of trimethylation of histone H3-lysine 27-trimethylated (H3K27me3) [11]. A previous study reported that microRNAs (miRNAs) are a series of molecules of non-coding RNA consisting of nearly 23 nucleotides, whose performance is essential to a mass of human diseases like depression [12]. Considerable quantity of miRNAs has been demonstrated to maintain proteostasis, support neurotrophy, and manipulate inflammation response, genetic intactness and transcriptional reaction in depression [13]. miR-29b-3p is downregulated in depression neurons and cortexes, which results in cellular death and cytodendrite deficiency and aggravated depression-like behaviors [14]. Besides, miR-29b expression is degraded upon microglia activation to encourage inflammatory responses and neuron loss [15]. A previous document has reported that miR-29b-3p is involved in the progression of pathological vascular calcification by targeting MMP2 [16]. MMP2 is robustly expressed in rats with depression [17]. MMP2 overexpression is accompanied with microglia activation, inflammatory reactions, and neuronal injuries [18]. Collectively, we made a hypothesis that EZH2 may influence neuroinflammation and microglia activation in depression via the regulation of miR-29b-3p/MMP2 axis. This experiment is designed to explore the role of EZH2 in inflammation of depression rats via microglia polarization, thus offering a therapeutic strategy for depression.

## Materials and methods

### Ethics statement

This study was approved by the ethics committee of Mental Medical College of Wenzhou Medical University. The protocol was also approved by the Institutional Animal Care and Use Committee of Mental Medical College of Wenzhou Medical University and *Guidelines for the Care and Use of Laboratory Animals* proposed by the National Institutes of Health.

### Establishment and treatment of depression rat model

A total of 24 adult male Sprague Dawley rats [180–200 g, SYXK (Beijing) 2017–0033, Beijing Vital River Laboratory Animal Technology Co., Ltd, Beijing, China] were raised in a laboratory animal center at 23 ± 2°C under 12-h light/dark cycles with 55 ± 10% humidity to acclimate for 7 days. Food and water were accessible to these rats. After 7 days of adaptive feeding, the rats were labeled for body weight and randomly assigned into the sham group, the chronic unpredictable mild stress (CUMS) group, the short hairpin (sh)-negative control (NC) group, and the sh-EZH2 group (N = 6 in each group).

Rat depression model was established via CUMS [19]. The applied items included (1) baking at 45°C for 5 min (rat in a separate cage was exposed to an electric heating lamp at 45°C for 5 min); (2) swimming in ice water at 4°C for 5 min; (3) 85 dB white noise stimulation for 5 h; (4) restraint stress for 3 h; (5) strange odor for 24 h (spraying glacial acetic acid on the litter for 24 h); (6) strange objects for 17 h (putting some plastic toys in the cage for 17 h) and (7) damp bedding for 17 h (putting in damp litter for 17 h). No same pressure was applied on any two consecutive days.

Rats were anesthetized with sodium pentobarbital (25 mg/kg) for the subsequent stereotaxic surgery. In brief, the rats were fixed in a stereotaxic frame and the hippocampal stereotaxic injection was conducted at 3.5 mm from the ventral surface of the skull, 2.5 mm from lateral to the medial suture and 4.8 mm behind the anterior fontanel. Matching catheter cores were inserted into the catheter to avoid obstruction. It took 42 days for CUMS model establishment. Then, 1 μL of sh-EZH2 or sh-NC (1 × 10^9^ viral genomes/μL, Shanghai GenePharma Co., Ltd, Shanghai, China) [20] was stereotaxically injected into the hippocampus of rats on the 35^th^ day of CUMS modeling at a constant rate within 1 min, and the needle was slowly retracted 5 min after injection. Rats in the sham group and CUMS group were given the same amount of normal saline. Penicillin sodium was injected intraperitoneally at 200,000 units per day for 3 consecutive days after lentivirus injection to prevent infection. Seven days after stereotaxic injection, all groups of rats were subjected to the behavioral tests.

### Sucrose preference test (SPT)

In SPT, the lack of pleasure in rats was assessed based on their preference for candies [21]. Briefly, two bottles with 1% sucrose (w/v) were placed in each cage for 24 h and one of them was then exchanged with drinking water. The position of bottles was changed every 2 h for the subsequent 24 h to avoid any possible positional preference. After adaptation, the rats were subjected to a 24-h food and water deprivation. The rats were then given one bottle of drinking water and 1% sucrose each for 2 h. Sucrose preference was calculated as sucrose consumption/(sucrose consumption + water consumption) × 100%.

### Open field test (OFT)

OFT was conducted for the assessment of anxiety level and autonomic activities as anxious rats typically spend less time staying at the central area [22]. Each rat was placed at the center of a 100 × 100 × 35 cm square field and observed using the Ethovision XT 11.5 video tracking system for 5 min to record the total distance travelled, average speed and frequency of feeding of rats. The apparatus was wiped with 75% ethanol after each test to remove any possible olfactory signals. The tests were carried out in a quiet room with a visibility of just 5 m.

### Forced swimming test (FST)

FST was carried out to test the depression-like symptoms of the rats exposed to different kinds of stressors [23]. During FST, the rats were forced to swim in a glass bottle (30 cm in diameter and 40 cm in height) with a water depth of 28 cm and a water temperature of 25°C for 6 min, with the last 4 min of immobility recorded. The immobility time of FST was regarded as the duration during which the rat stopped struggling in the water to float or only showed a slight exposure of their heads above the water.

### Cell culture

Primary microglia were obtained from the brains of rats at 1 to 2 days postnatal [24]. The brains were carefully removed under aseptic conditions to isolate the cerebral cortex and excise the meninges. The cortices were then gently separated and filtered through a 70 μm nylon cell strainer (BD Biosciences, Heidelberg, Germany). Cells were centrifuged at 1000 *g* for 10 min, collected, suspended in Dulbecco’s modified Eagle’s medium (DMEM, Sigma-Aldrich, Merck KGaA, Darmstadt, Germany) with 10% fetal bovine serum (Gibco Company, Grand Island, NY, USA), 40 U/mL penicillin and 40 μg/mL streptomycin (both from Sigma), and cultured in the cell culture dishes (10 cm, Falcon, Heidelberg, Germany) at a density of 5 × 10^5^ cells/mL at 37°C with 5% CO_2_. The complete DMEM was refreshed every 24 h and then every 3 days. After 12 days of *in vitro* culture, DMEM was gently and physically shaken to extract the visibly floating microglia, which were then re-seeded in the cell culture plates according to the experimental design. DMEM was changed every other day to discard non-adherent cells, and the cells were stimulated 1 h later for the corresponding experiments.

### Cell treatment and grouping

sh-EZH2 or sh-NC (2.8 × 10^8^ TU/mL) was appointed to infect microglia. miR-29b-3p inhibitor or NC-inhibitor (RiboBio Co., Ltd, Guangzhou, Guangdong China) was transfected into microglia using Lipofectamine 2000 (Invitrogen Inc., Carlsbad, CA, USA) for further analysis. After the transfection, microglia were incubated in the medium with 100 ng/mL lipopolysaccharide (LPS) for 24 h [25].

### Immunofluorescence

After behavioral tests, the rats were anaesthetized with sodium pentobarbital (50 mg/kg), and their limbs were fixed. Next, cold saline and 4% paraformaldehyde were instilled intracardially into the rats. The brain was carefully removed and the hippocampus was quickly dissected for biochemical experiments. Hippocampus was fixed in 4% paraformaldehyde for 24 h at 4°C, dehydrated, cleared, paraffin-embedded, and made into sections (5 μm). Next, the sections were then dewaxed and hydrated for antigen extraction. After 3 washes in phosphate buffer saline (PBS), the sections were blocked with 5% bovine serum albumin in PBS for 30 min and then incubated with an primary antibody ionized calcium binding adaptor molecule 1 (Iba-1) (ab178846, Abcam Inc., Cambridge, MA, USA) at 4°C overnight. After PBS washing, the sections were incubated with secondary antibody goat anti-rabbit immunoglobulin G (IgG) (ab150077, Abcam) at 37°C for 30 min. After 3 PBS washes, the sections were treated with streptavidin-biotin complex at 37°C for 30 min and washed 3 times with PBS again. Next, the nucleus was stained using 4’, 6-diamidino-2-phenylindole (DAPI). Images of the sections were obtained with a camera attached to an Olympus microscope. The number of positive cells was calculated using the ImageJ software 1.50i (NIH, Bethesda, MD, USA), and the results were expressed as number of positive cells per mm^2^ [26].

As previously described [27], the cultivated cells or microglia with different treatments were fixed in 4% paraformaldehyde for 30 min at room temperature, rinsed with PBS 3 times, treated by 0.3% Triton X-100 for 15 min, blocked by serum for 30 min, and cultured with primary antibody rabbit anti-iba-1 (ab178846, Abcam) overnight, followed by incubation with goat anti-rabbit IgG H&L (Alexa Fluor® 488) (ab150077, Abcam) for 1 h. Additionally, nucleus stained using DAPI turned into blue. Then, cells were observed under a fluorescence microscope (Olympus).

### Chromatin immunoprecipitation (ChIP) assay

ChIP assay was performed with the assistance of the ChIP assay kit (Thermo Fisher Scientific, Waltham, MA, USA) [11]. The hippocampal tissue was cut into small pieces and crosslinked in 1.5% formaldehyde, and added with 0.125 M glycine to terminate the crosslinking. Next, the tissue pieces were centrifuged at 1000 g at 4°C for 5 min, and the culture medium was absorbed; the tissue pieces were washed with cold PBS, centrifuged at 1000 g at 4°C for 5 min again, and the cleaning buffer was discarded; subsequently, the tissue pieces were resuspended in cold PBS to prepare the cell suspension, and resuspended with lysis buffer. Microglia (1.5 × 10^7^) were cross-linked in 1% formaldehyde and subjected to sonication and shearing using Bioruptor Plus (Diagenode, Denville, NJ, USA). Chromatin was immunoprecipitated (incubated overnight at 4°C) with EZH2 (PA5-17569, Thermo Fisher) and H3K27me3 (ab192985, Abcam) or rabbit IgG (as the NC, ab133470, Abcam). Chromatin extracts were incubated with 20 μL ChIP-level protein A/G plus agarose at 4°C for 3 h and centrifuged at 12,000 *g* for 15s to collect the combined agarose beads, which were rinsed to elute the precipitated protein-DNA complexes. The complexes were incubated twice at 65°C for 1.5 h with NaCl and Proteinase K to reverse cross-links. The purified DNA was subjected to reverse transcription quantitative polymerase chain reaction (RT-qPCR) using GoTaq qPCR Master Mix (Promega, Madison, WI, USA).

### Dual-luciferase reporter gene assay

The interaction between miR-29b-3p and MMP2 was predicted via the Targetscan database (www.targetscan.org) [28] and then verified using the dual-luciferase reporter gene assay kit (Promega) [25]. HEK293 cells (American Type Culture Collection, Manassas, VI, USA) were seeded in 96-well plates, and transfected with 100 ng pGL3-MMP2-wild type (WT)/pGL3-MMP2-mutant type (MUT) and miR-29b-3p mimic or mimic NC using Lipofectamine 2000 (Invitrogen). Luciferase activity was determined using the dual-luciferase assay system 48 h later.

### Enzyme-linked immunosorbent assay (ELISA)

The hippocampus undergoing different treatments were homogenized using PBS and centrifuged at 8000 *g* at 4°C for 15 min to harvest the supernatant. Afterwards, the levels of M1 pro-inflammatory cytokines interleukin (IL)-1β (ab255730) and tumor necrosis factor-α (TNF-α) (ab236712), and M2 anti-inflammatory cytokines IL-10 (ab214566) and IL-4 (ab100771, all from Abcam) were analyzed using the specific ELISA kit and calculated according to the standard curve [21].

### RT-qPCR

The total RNA was extracted from the hippocampus or microglia using the TRIzol reagent (Thermo Fisher) [19]. Total RNA concentration was then determined using a spectrophotometer (Eppendorf, Hamburg, Germany) and RNA purity was assessed using 1% agarose gel electrophoresis. The RNA was synthesized into the first-strand complementary DNA (cDNA) using the revert aid first strand cDNA synthesis kit (Thermo Fisher) via C1000 Touch^TM^ Thermal Cycler (Bio-Rad, CA, USA). Next, the cDNA was amplified using the SYBR green PCR master mix (Thermo Fisher) on the multicolor real-time fluorescent qPCR assay system (Bio-Rad Laboratories Inc., Hercules, CA, USA) with the following cycling parameters: at 95°C for 10 min, then 40 cycles of at 95°C for 15s, 55°C for 1 min, 65°C for 5s, and 95°C for 15s. Glyceraldehyde-3-phosphate dehydrogenase was used as an internal reference for mRNA and U6 as an internal reference for miRNA, and the relative expression was calculated using the 2^−ΔΔCt^ method [29]. The primers used are shown in Table 1.

**Table 1.** Primer sequence of RT-qPCR

Name of primer	Sequences (5’-3’)
EZH2	F: ATGGGCCAGACTGGGAAGAAATCTG
	R: TCAAGGGATTTCCATTTCTCGTTC
miR-29b-3p	F: TAGCACCATTTGAAATCAGTGTT
	R: AACACTGATTTCAAATGGTGCTA
MMP2	F: ATGGAGGCACGATTGGTCTGGGGA
	R: TCAGCAGCCCAGCCAGTCCGATT
iNOS	F: ATGGCTTGCCCCTGGAAGTTTCT
	R: TCAGAGTCTTGTGCCTTTGGGCTC
CD16	F: ATGTGGCACCTACTACTACCAAC
	R: TCACTTGTCCTGAGGGTCCTTGCT
Arg-1	F: ATGAGCTCCAAGCCAAAGCCCATA
	R: TTATTTCGGTGGTTTAAGGTAGT
CD206	F: ATGAGACTCCCCCTGCTCCTGG
	R: CTAAATGACCGCATGCTCATTC
GAPDH	F: ATGGTGAAGGTCGGTGTGAACGG
	R: TTACTCCTTGGAGGCCATGTAGG
U6	F: ATGGCGGACGACGTAGATCAGCA
	R: TCAGCCAACTCTCAATGGAGGGG

RT-qPCR, reverse transcription-quantitative polymerase chain reaction; EZH2, enhancer of zeste homolog 2; miR, microRNA; MMP2, matrix metallopeptidase 2; iNOS, inducible nitric oxide synthase; Arg, argininase; GAPDH, glyceraldehyde-3-phosphate dehydrogenase; F, forward; R, reverse

### Western blot analysis

The hippocampus or microglia were collected and lysed in radio-immunoprecipitation assay buffer (Beyotime Biotechnology Co., Ltd, Shanghai, China) for the extraction of total protein [21]. The protein content was determined by bicinchoninic acid kits (Beyotime). Proteins were loaded onto 7.5% sodium dodecyl sulfate-polyacrylamide gel electrophoresis and then transferred onto polyvinylidene fluoride membranes (Millipore, Billerica, MA, USA). Membranes were sealed with 5% skim milk for 1 h and incubated with primary antibodies: EZH2 (1:1000, PA5-17569, Thermo Fisher), inducible nitric oxide synthase (iNOS) (1:1000, PA1-036, Thermo Fisher), argininase (Arg)-1 (1:5000, PA5-29645, Thermo Fisher), H3K27me3 (1:1000, ab192985, Abcam), and β-actin (ab8227, 1:1000, Abcam) at 4°C overnight, followed by incubation with horseradish peroxidase-coupled secondary antibody (1:2000, ab6721, Abcam) for 2 h at room temperature. The positive bands were visualized with enhanced chemiluminescence assay reagent (Thermo Scientific). The optical density value of bands was measured using BioRad gel imager and Image J software (Bio-Rad). The relative expression of proteins was analyzed using Image J software (National Institutes of Health, Bethesda, MD, USA), with β-actin as an internal reference.

### Statistical analysis

SPSS 21.0 software (IBM Corp. Armonk, NY, USA) was appointed for data analysis and GraphPad Prism 8.0 software (GraphPad Software Inc., San Diego, CA, USA) was used for graphing. All data were inspected with normality distribution and homogeneity test of variance. The *t*-test was appointed for comparison analysis between two groups and one-way or two-way analysis of variance (ANOVA) was appointed for comparison analysis among multiple groups, and Tukey’s multiple comparisons test or Sidak’s multiple comparisons test was used for post-test of data. The *p* value was attained using a two-tailed test and a value of *p* < 0.05 indicated a significant difference.

## Results

The main purpose of this paper is to explore the effect and mechanism of histone methyltransferase EZH2 on neuroinflammation in depression rats. We speculate that the miR-29b-3p/MMP2 axis is a potential downstream mechanism of EZH2. We verified the protective effect of silencing EZH2 on depression rats through *in vivo* and *in vitro* models, and found that EZH2 played an important role in regulating microglia polarization. In addition, we found that EZH2-mediated H3K27me3 modification promoted MMP2 transcription by inhibiting the expression of miR-29b-3p. Our research focuses on the role and mechanism of EZH2 in depression, which provides a new direction for the treatment of depression.

### EZH2 is strongly expressed in depression rats

**Figure 1.**
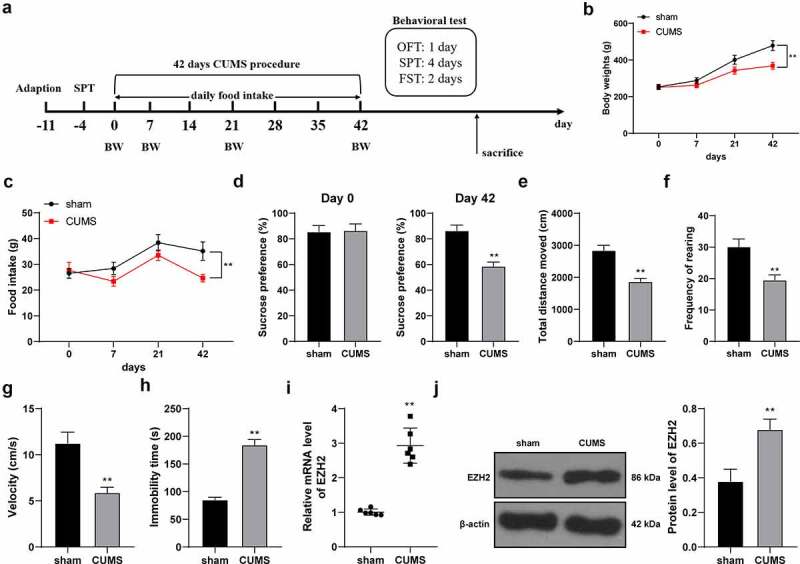
EZH2 is strongly expressed in depression rats. (a) Rat depression model established via CUMS treatment. (b) Body weight of rats with CUMS induction. (c) Food intake of rats with CUMS induction. (d–h) Depression-like behaviors observed using SPT (d), FST (E, F and G) and OFT (h). I and J, EZH2 expression in the hippocampus assessed by RT-qPCR (i) and Western blot analysis (j). N = 6. The results in panels B, C, D, E, F, G, H and J were presented as mean ± standard deviation. Two-way ANOVA was used to analyze the data in panels B and C. Sidak’s multiple comparisons test was applied for post hoc test. The *t*-test was used to analyze the data in panels D, E, F, G, H, I and J. ** *p* < 0.01. BW: body weight; CUMS: chronic unpredictable mild stress; SPT: sucrose preference test; OFT: open field test; FST: forced swimming test.

EZH2 is highly expressed in depression rats [10], but its effect and mechanism in depression rats are yet to be elucidated. To this end, depression rat model was established by CUMS treatment (Figure 1(a)) and CUMS-induced behavioral changes were observed using SPT, FST and OFT. After modeling, the food intake and body weight of CUMS-treated rats were lower than those of sham-operated rats (*p* < 0.01, Figure 1(b,c)). Sucrose preference (*p* < 0.01, Figure 1(d)), total walking distance (*p* < 0.01, Figure 1(e)), feeding frequency (*p* < 0.01, Figure 1(f)) and mean speed (*p* < 0.01, Figure 1(g)) of CUMS-treated rats were declined. Compared with the sham group, the CUMS group showed increased immobility time in the FST experiment (*p* < 0.01, Figure 1(h)). The above results suggested that the depression rat model was successfully established. In addition, we discovered that EZH2 expression in the hippocampus of CUMS-treated rats was higher than that in the sham-operated rats (*p* < 0.01, Figure 1(i,j)).

### EZH2 silencing reduces the depression-like behaviors of depression rats

**Figure 2.**
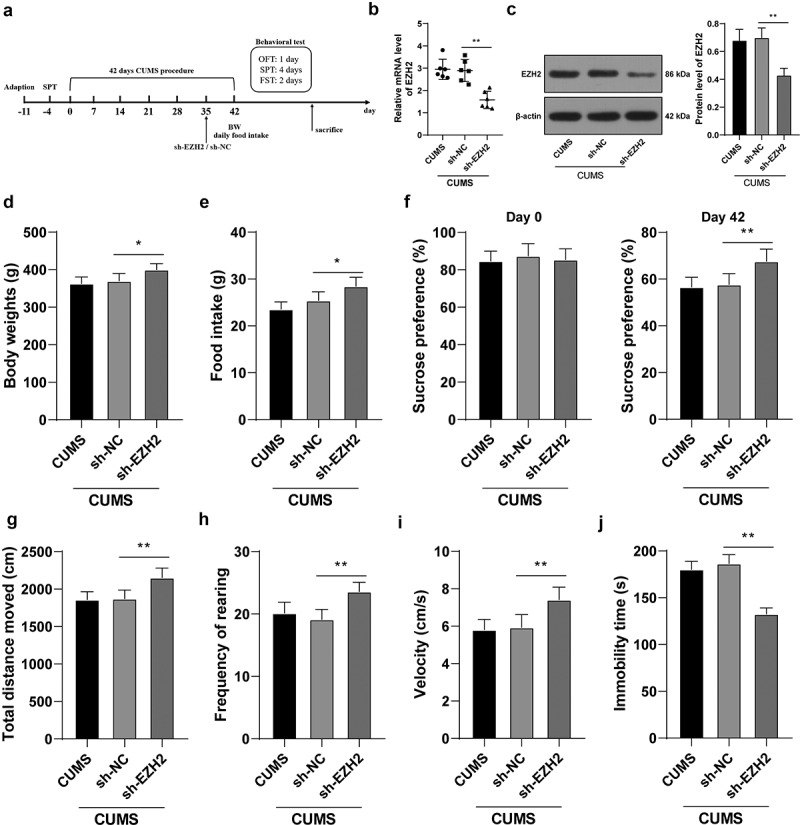
EZH2 silencing reduces the depression-like behaviors of depression rats. sh-EZH2 was injected into the hippocampus of CUMS-treated rats, with sh-NC injection as the control. (A) sh-EZH2 injection into the hippocampus of CUMS-treated rats. (b,c) mRNA and protein levels of EZH2 determined by RT-qPCR and Western blot analysis. (d) Food intake of rats undergoing CUMS induction. (e) Body weight of rats during CUMS induction. (f–j) depression-like behaviors observed using SPT (f), FST (g-i) and OFT (j). N = 6. The results were presented as mean ± standard deviation. One-way ANOVA was used to analyze the data. Tukey’s multiple comparisons test was applied for post hoc test. * *p* < 0.05, ** *p* < 0.01.

To verify the role of EZH2 in depression rats, EZH2 levels in the hippocampus of CUMS-treated rats were successfully reduced (*p* < 0.01, Figure 2(a)) through the injection of sh-EZH2 into the hippocampus (Figure 2(b,c)). Subsequently, the results showed that the body weight and food intake of CUMS-treated rats were increased upon EZH2 silencing (*p* < 0.05, Figure 2(d,e)). When EZH2 expression was decreased, sucrose preference, total walking distance, feeding frequency, and mean speed were all elevated (all *p* < 0.01, figure 2(f–i)) and immobility time was reduced (*p* < 0.01, Figure 2(j)) in CUMS-treated rats. Altogether, EZH2 silencing helps to recover from the depression-like behaviors in depression rats.

### EZH2 silencing palliates neuroinflammation in depression rats by regulating microglia polarization

**Figure 3.**
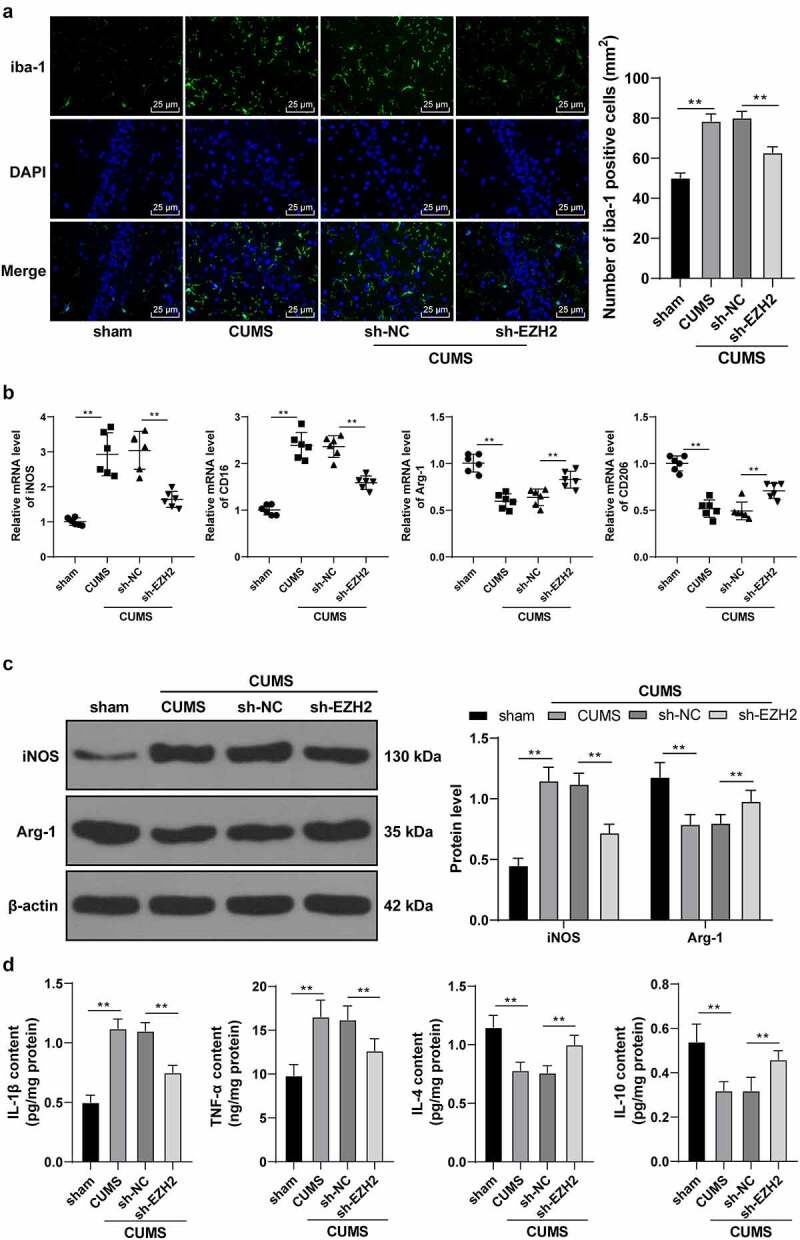
EZH2 silencing palliates neuroinflammation in depression rats by regulating microglia polarization. (a) Positive rate of iba-1 analyzed by immunofluorescence. (b) mRNA levels of iNOS, CD16, Arg-1 and CD206 detected by RT-qPCR. (c) Protein levels of iNOS and Arg-1 determined by Western blot analysis. (d) Contents of IL-1β, TNF-α, IL-4 and IL-10 measured by ELISA. N = 6. The results in panels A, C and D were presented as mean ± standard deviation. One-way ANOVA was used to analyze the data in panels A, B and D. Two-way ANOVA was used to analyze the data in panel C. Tukey’s multiple comparisons test was applied for post hoc test. ** *p* < 0.01.

EZH2 regulates microglia activation and plays a principal role in the expression of pro-inflammatory cytokines [10]. A growing number of studies have shown that high M1/M2 ratio in microglia is associated with neurological deficits and depression in rats [30,31]. Our findings unraveled a significant increase in the positive rate of iba-1 in the CUMS group while the sh-EZH2 group showed reduced iba-1 positive rate (*p* < 0.01, Figure 3(a)). Results of RT-qPCR found that CUMS induction promoted mRNA levels of M1-type markers iNOS and CD16, but reduced mRNA levels of M2-type markers Arg-1 and CD206; while EZH2 silencing led to opposite trends (*p* < 0.01, Figure 3(b)). Likewise, results of Western blot analysis showed a same trend as those of the RT-qPCR did (*p* < 0.01, Figure 3(c)). Besides, levels of relevant inflammatory factors were examined, which revealed that CUMS treatment elicited inflammatory response in rats (increase in the levels of M1 pro-inflammatory factors IL-1β and TNF-α, and decrease in the levels of M2 anti-inflammatory factors IL-4 and IL-10), while EZH2 silencing reduced inflammatory symptom in CUMS-treated rats (*p* < 0.01, Figure 3(d)). Altogether, EZH2 silencing palliated neuroinflammation in depression rats by eliciting microglia M2-type polarization.

### EZH2 silencing triggers LPS-induced microglia polarization

**Figure 4.**
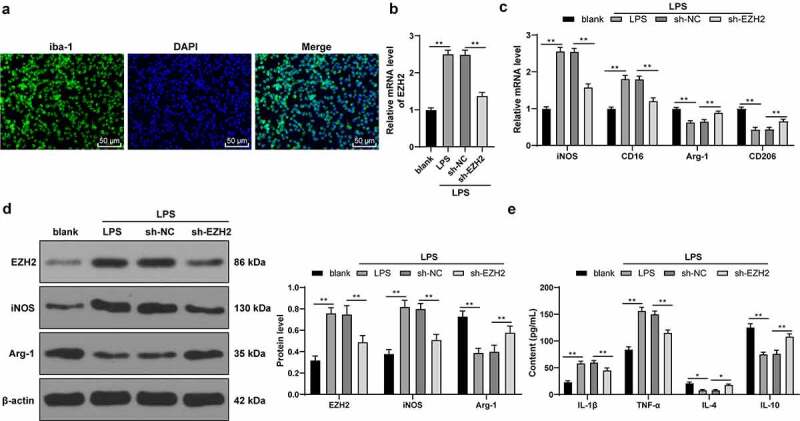
EZH2 silencing triggers M2 polarization of LPS-induced . (a) Positive rate of iba-1 in LPS-induced microglia analyzed by immunofluorescence. (b) mRNA levels of EZH2 detected by RT-qPCR. (c) mRNA levels of iNOS, CD16, Arg-1 and CD206 detected by RT-qPCR. (d) Protein levels of EZH2, iNOS and Arg-1 determined by Western blot analysis. (e) Contents of IL-1β, TNF-α, IL-4 and IL-10 measured by ELISA. The experiment was performed 3 times. The results were presented as mean ± standard deviation. One-way ANOVA was used to analyze the data in panel B. Two-way ANOVA was used to analyze the data in panels C, D and E. Tukey’s multiple comparisons test was applied for post hoc test. * *p* < 0.05, ** *p* < 0.01.

Subsequently, the effect of EZH2 was elaborately validated *in vitro*. Rat microglia were isolated and cultured for immunofluorescence detection, which found that the cultured microglia had a positive rate of iba-1 over 95% (Figure 4(a)). Next, microglia were activated with LPS, and EZH2 levels were upregulated upon LPS treatment (*p* < 0.01, Figure 4(b,d)). Next, EZH2 levels were successfully declined in LPS-induced cells via the sh-EZH2 injection into the cells (*p* < 0.01, Figure 4(b,d)). Furthermore, LPS treatment increased expressions of iNOS and CD16 and decreased expressions of Arg-1 and CD206; whereas EZH2 silencing inhibited the effect of LPS (*p* < 0.01, Figure 4(c,d)). In addition, LPS treatment increased inflammatory levels in microglia; while EZH2 silencing reversed the promotion in inflammation (*p* < 0.05, Figure 4(e)). The above findings indicated that EZH2 silencing induced M2-type polarization of LPS-induced microglia.

### EZH2 targets miR-29b-3p to promote MMP2 transcription

**Figure 5.**
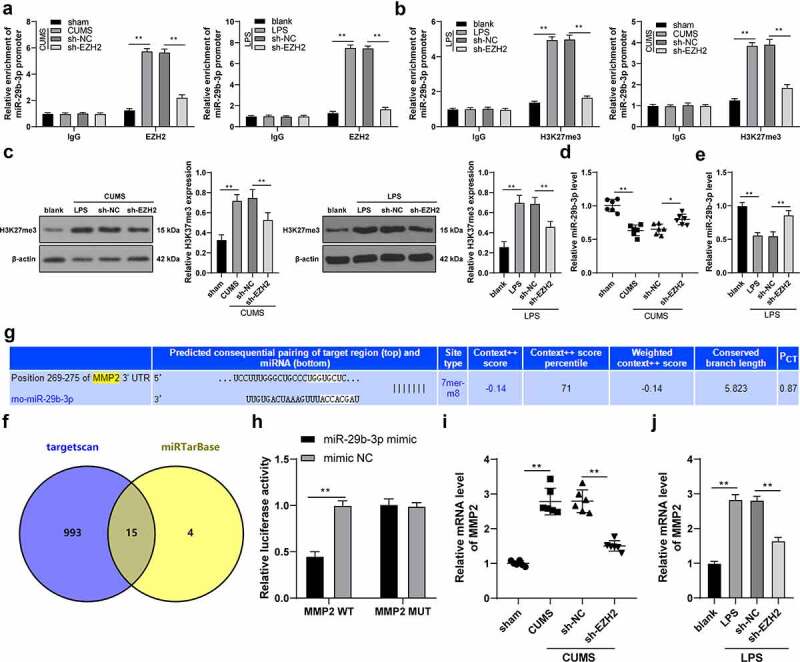
EZH2 targets miR-29b-3p to promote MMP2 transcription. (a,b) The binding relation between EZH2 and miR-29b-3p verified by ChIP assay. (c) H3K27me3 level detected by Western blot analysis. (d,e) The level of miR-29b-3p in depression rats and LPS-induced microglia measured by RT-qPCR. (f) the downstream target genes of miR-29b-3p predicted by Targetscan database (http://www.targetscan.org/vert_71/) and miRTarBase (http://mirtarbase.cuhk.edu.cn/php/search.php), with the intersections obtained. (g) The binding sites between miR-29b-3p and MMP2. (h) The binding relation between miR-29b-3p and MMP2 validated by dual-luciferase reporter gene assay. (i,j) Transcription level of MMP2 in hippocampus (i) and microglia (j). N = 6. The experiment was performed 3 times. The results in panels A-C, E, H and J were presented as mean ± standard deviation. Two-way ANOVA was used to analyze the data in panels A-B and H. Sidak’s multiple comparisons test was applied for post hoc test. One-way ANOVA was used to analyze the data in panels C-E and I-J. Tukey’s multiple comparisons test was applied for post hoc test. * *p* < 0.05, ** *p* < 0.01.

ChIP assay unveiled that EZH2 could bind to the promoter region of miR-29b-3p; after silencing EZH2, the enrichment of EZH2 on the miR-29b-3p promoter was decreased (*p* < 0.01, Figure 5(a)). After modeling, the H3K27me3 level was increased in tissues and cells and the enrichment of H3K27me3 on the miR-29b-3p promoter was increased, while EZH2 silencing resulted in reversed trends (*p* < 0.01, Figure 5(b,c)). Besides, miR-29b-3p was weakly expressed in depression rats and in LPS-induced cells, but increased upon EZH2 silencing (*p* < 0.05, Figure 5(d,e)). Next, the downstream target genes of miR-29b-3p were predicted in the Targetscan database (http://www.targetscan.org/vert_71/) and miRTarBase (http://mirtarbase.cuhk.edu.cn/php/search.php) to obtain the intersections, and eventually 15 target genes were observed (Figure 5(f)), among which MMP2 was highly expressed in depression patients and correlated with microglia activation [17,18]. The binding relationship between miR-29b-3p and MMP2 was confirmed by the binding sites (Figure 5g) and dual-luciferase reporter gene assay (*p* < 0.01, Figure 5(h)). Moreover, the transcription level of MMP2 was found to increase in both *in vivo* and *in vitro* models, while it was decreased upon EZH2 silencing (*p* < 0.01, Figure 5(i,j)). In summary, EZH2 represses miR-29b-3p expression by binding to the promoter of miR-29b-3p and upregulating H3K27me3 methylation level, thereby promoting MMP2 transcription.

### Inhibition of miR-29b-3p expression mitigates the role of EZH2 silencing in LPS-induced microglia polarization

**Figure 6.**
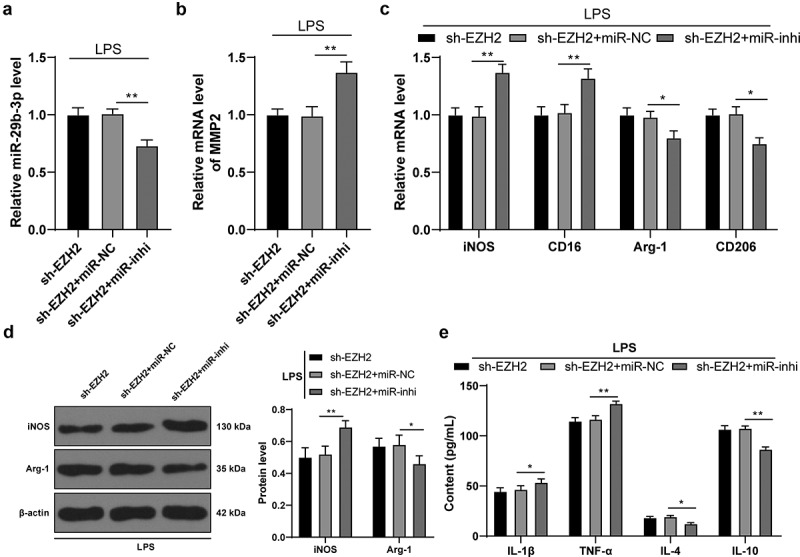
Inhibition of miR-29b-3p expression mitigates the role of EZH2 silencing in LPS-induced microglia polarization. miR-29b-3p inhibitor was transfected into the microglia in the sh-EZH2 group, with the sh-NC as the control. (a) miR-29b-3p expression assessed by RT-qPCR. (b) Transcription levels of MMP2 detected by RT-qPCR. (c) mRNA levels of iNOS, CD16, Arg-1 and CD206 detected by RT-qPCR. (d) Protein levels of iNOS and Arg-1 determined by Western blot analysis. E, contents of IL-1β, TNF-α, IL-4 and IL-10 measured by ELISA. The experiment was performed 3 times. The results were presented as mean ± standard deviation. One-way ANOVA was used to analyze the data in panels A and B. Two-way ANOVA was used to analyze the data in panels C, D and E. Tukey’s multiple comparisons test was applied for post hoc test. * *p* < 0.05, ** *p* < 0.01.

To verify the role of miR-29b-3p in depression, miR-29b-3p inhibitor was transfected into cells in the sh-EZH2 group to successfully reduce intracellular miR-29b-3p expression (*p* < 0.01, Figure 6(a)). Inhibition of miR-29b-3p brought about a subsequent increase of MMP2 transcription level (*p* < 0.01, Figure 6(b)), a polarization of microglia mainly towardsM1-type (*p* < 0.05, Figure 6(c,d)) and an increased inflammatory level (*p* < 0.05, Figure 6(e)). The above results suggested that inhibition of miR-29b-3p mitigated the role of EZH2 silencing on LPS-induced microglia polarization.

## Discussion

Depression refers to impairment emerged from biased mood and thoughts, which is evidenced as the extremely negative view towards oneself, future and even the world [2]. Neuroinflammation and microglia activation are the major reasons of mitochondrial disruption and neuronal damage in depression [32]. Histone methylation modulates RNA expression variance, so as to affect the molecular and cellular pathways in brains with depression [33]. EZH2, a histone methyltransferase, is critical in the process of cellular development, immune system and neuronal function of psychiatric diseases [34]. EZH2 depletion contributes to suppressed inflammatory response and depression-like behaviors in obese depression mice [35]. Our study pointed out the effect of EZH2 in neuroinflammation and microglia activation of depression with the participation of the miR-29b-3p/MMP2 axis.

It was documented in a recent report that in depression rat challenged by CUMS, EZH2 expression was promoted [5]. In our experiments, depression rat model was established via the CUMS treatment, after which we observed that EZH2 was robustly expressed in depression. Consistently, EZH2 expression was also increased in subjects harassed from anxiety [36]. To verify the role of EZH2 in depression rats, mRNA level of EZH2 in the hippocampus of CUMS-treated rats was reduced through the injection of sh-EZH2 into the hippocampus. Sucrose preference, total walking distance, feeding frequency and mean speed of CUMS-treated rats were declined after EZH2 inhibition. Consistently, the inhibition of EZH2 was associated with improved cognitive function and relieved depression-like behaviors [37]. Briefly, EZH2 silencing helps depression rats to recover from the depression-like behaviors.

There are mainly two types of microglia polarization, which is categorized as M1 (pro-inflammatory effect) and M2 (anti-inflammatory effect), and M1-type was intensified during psychiatric disorders while M2-type could prevent neurons from inflammatory damage [8]. Previous study has suggested that microglia polarization is vital in the pathogenesis of major depressive disorder [38]. EZH2 silencing evicted iNOS recruitment in rodents and human cells [39]. Inflammatory reaction and depression could motivate each other, as inflammation could be exaggerated by stressors-induced by depression whereas depression is further promoted by the ceaseless release of inflammatory cytokines [40]. Neuroinflammation encourages microglia activation to recruit stressors in hippocampus of brain from individuals with psychiatric illness, including depression [41]. Subsequently, our results showed that EZH2 silencing palliated neuroinflammation in depression rats by regulating microglia polarization as presented by the reduced iba-1 positive rate, decreased mRNA levels of M1-type markers iNOS and CD16, upregulated mRNA levels of the M2-type markers Arg-1 and CD206, declined levels of M1 pro-inflammatory factors IL-1β and TNF-α, and elevated levels of M2 anti-inflammatory factors IL-4 and IL-10. Sudden or abnormal activation or polarization of microglia triggered by inflammatory infiltration or stressor exposure could be the culprit of neuronal dysfunction and neuroplasticity damage in depression [42]. Similarly, the activation of liver X receptors significantly prevented emotional and cognitive deficits induced by CUMS or LPS, prevented the up-regulation of inflammatory factors, and inhibited microglial M1-polarization [43]. EZH2 knockout was responsible for a cascade of variances in neuroinflammation, such as downregulated levels of IL-1β and TNF-α and upregulated level of IL-10 [44]. In that sense, EZH2 silencing could retard depression by suppressing inflammatory reaction and microglia activation. Furthermore, the effect of EZH2 was also studied in the cell level. Rat microglia were isolated, cultured and activated with LPS, and EZH2 mRNA level was upregulated upon LPS treatment. Next, EZH2 expression was declined in LPS-induced cells via the sh-EZH2 injection into the cells. The *in vitro* experiments supported that EZH2 silencing induced M2-type polarization of LPS-induced microglia.

Histone methylation is tightly related to depression progression [45]. The destructive role of overexpressed EZH2 in promoting H3K27me3 enrichment was repeatedly highlighted in studies related to neuronal damages [46,47]. Furthermore, the mechanism of EZH2 cooperating with H3K27me3 to modulate the downstream target miR expression and alteration could mediate cellular viability, development and death [48]. miRNAs are able to identify mental disorders including schizophrenia and depression by the transferring into microglia, neurons and oligodendrocytes of brains to inhibit specific cytokines [49]. Some studies reported the role of miRNAs in depression [50] and the effect of drug (Paeoniflorin) treatment on depression [51], but there was no report on the role of histone methyltransferase in depression. miR-29b expression was downregulated in brain tissues of depression patients [52]. In the present study, LPS induction increased the level of H3K27me3 in cells, while EZH2 silencing resulted in a reversed trend. Besides, miR-29b-3p was weakly expressed in depression rats and in LPS-induced cells, but increased upon EZH2 silencing. To elaborately verify the role of miR-29b-3p in depression, miR-29b-3p inhibitor was transfected into the sh-EZH2-treated cells to reduce intracellular miR-29b-3p expression, and it was unveiled that both inflammatory response and microglia polarization into M1 type were enhanced. In cells stimulated by LPS, miR-29b-3p was weakly expressed, whereas its overexpression significantly sabotaged inflammatory symptoms [53]. Moreover, miR-29b protected organ damage by suppressing iNOS [54]. miR-29b was poorly expressed in airway inflammation by strengthening pro-inflammatory cytokine levels and it predicted impaired organ functions [55]. Essentially, our findings unraveled that miR-29b-3p could target MMP2. mRNA level of MMP2 was found to increase in both *in vivo* and *in vitro* models, while it was decreased upon EZH2 silencing. MMP2 derived from neuronal damage accelerated microglia activation, thereby unleashing inflammatory cytokines [56]. Furthermore, MMP2 functioned as a valuable biomarker and a possible reason for depression as it was consistently overexpressed in depression [57]. Collectively, we may draw a conclusion that EZH2 knockdown could alleviate neuroinflammation and promote microglia M2 polarization by regulating the miR-29b-3p/MMP2 axis.

## Conclusion

In conclusion, our data supported that EZH2 targeted miR-29b-3p expression by promoting H3K27me3 level to elevate MMP2 transcription and trigger microglia M1-type polarization, so as to exacerbate depression-like behaviors and neuroinflammation in depression rats. These results discovered a therapeutic implication for depression treatment. In the future, we will further explore the underlying mechanism involved in depression and the potential therapeutic options. Still, this is just a preclinical research with some limitations. For example, we only verified the effect of EZH2 on microglia polarization, whether there are other functions of EZH2 remains to be investigated. The other downstream genes of EZH2 and miR-29b-3p should also be figured out. There is overlap between the downstream target genes of miR-29a-3p, miR-29c-3p and miR-29b-3p. We have not explored the expression of miR-29a-3p and miR-29c-3p in depression, nor have we explored that the expression changes of miR-29a-3p and miR-29c-3p can affect the transcription level of MMP2. It is also unclear whether miR-29c-3p and miR-29b-3p jointly affect the transcriptional level of MMP2. The experiment results and effective application into clinical practice need further validation. It still has a long way to go before it is widely applied in clinical practice. And we hope our experiment could contribute some implications to the depression research.
